# Behavioral and Social Considerations

**DOI:** 10.1093/geroni/igaa057.3147

**Published:** 2020-12-16

**Authors:** Lisa Barry

**Affiliations:** UConn Center on Aging, Farmington, Connecticut, United States

## Abstract

Cognitive, behavioral and social dimensions also demonstrate increasing heterogeneity with aging. For example, a longitudinal study of over 1,000 clergy revealed increasing heterogeneity in cognitive function and rate of decline with aging. Moreover, studies of individuals with probable Alzheimer’s disease have shown heterogeneity in terms of clinical manifestations and rates of cognitive decline. Older adults also demonstrate greater heterogeneity in mood, anxiety, and the nature and patterns of symptoms over time. Heterogeneity of overall health status increases with aging, as does reported quality of life. Health and Retirement Study (HRS) data have shown that low socioeconomic status or being an underrepresented minority are both associated with greater intra-individual variability in health status in old age, with greatest differences seen in Hispanics. Finally, early life adversity can contribute to heterogeneity of multidimensional health trajectories even in late life.

